# Lectures on Natural and Difficult Parturition

**Published:** 1847-12

**Authors:** 


### BIBLIOGRAPHICAL NOTICES.

---

ART. VII.—*Lectures on Natural and Difficult Parturition.* By EDWARD WILLIAM MURPHY, A.M., M.D., Professor of Midwifery, University College, London; Obstetric Physician, University College Hospital; and formerly Assistant Physician to the Dublin Lying-in Hospital. New York: Samuel S. and William Wood.

A press of duties has prevented us from giving, until now, the lengthened notice of this work to which its merits entitle it, and though late, we return to it at this time for the purpose of presenting our readers with such an analysis of its matter, as will enable them to judge whether the very favorable opi nion which we expressed concerning it, on its first appearance, is too high. The author, besides being a teacher of eminence in this branch of the profession, is possessed of social and moral qualities which impart an additional interest to his lectures for those who enjoy his acquaintance. His attentions to our young countrymen have made him friends, on this side the Atlantic, who will always rejoice in his prosperity, and receive with a liberal spirit whatever may emanate from his pen. Apart from any prepossessions in his favor created by this cause, the intrinsic excellence of these lectures ought to insure them an extensive circulation. We are sure that no one can rise from their perusal without a conviction, that they are the offspring of a strong, practical, cultivated mind. The volume comprising them is not large, and yet it will be found to evince research on all the points presented. Passing over the two first lectures in which the anatomy of the Pelvis is described, our first extract is furnished by the succeeding one on the *mechanism of parturition*—a subject, as our readers all know, upon which obstetricians are far from being of one mind.

"We would now direct your attention to the means provided by nature, to prevent the danger which might arise during this process. If the uterus exerted its full power upon the undilated os uteri, and if the unyielding head of the child were driven forcibly against it, the almost certain consequence would be, that the irritation would excite increased resistance, and ultimately terminate in inflamation of the mouth of the uterus. To obviate such an effect, nature interposes *a fluid medium* between the power and the resistance. The liquor amnii contained within the membranes, occupies the cavity of the uterus, and when its parietes contract upon it, the force exerted is (as we have explained) by this means, accurately conveyed to the os uteri. When the latter dilates in the slightest degree, the fluid insinuates itself within the smallest opening, and expands it by a direct lateral pressure against its edges. The power of the uterus is thus made to act in the most favorable manner for distending its mouth.

"Other advantages are also gained. The os uteri may dilate irregularly; but any attempt to overcome forcibly the undilated portion, is prevented when the force is conveyed through a fluid, which, while it readily yields to an undue resistance, still maintains an equable pressure upon the edges of the os uteri. Any irregularity in the action of the uterine fibres, is also, to a certain extent, obviated, because these contractions, although irregular, being still conveyed by the fluid, are thus equally communicated to the os uteri. Further, so long as the tissue of the uterus intervenes, it is necessary to moderate the great power which the uterus is capable of exercising to dilate it: this is effected by the liquor amnii. The force conveyed by a fluid, you are aware, does not act in one direction only, but is distributed to every part of the surface to which the fluid is applied. The force, therefore, which is exerted to expand the mouth of the uterus, being communicated by a fluid, is not only directed against the os tincæ, but against the fundus and sides of that organ. The fundus, consequently, is opposed, not only by the os uteri, but by its own action reflected by the liquor amnii. Hence, so long as the fluid remains and the os uteri is undilated, the more powerful the action of the fundus, the greater is the resistance to it. The actual force employed, is therefore very moderate, and any sudden or violent effort at distension is altogether obviated. You may observe this in the character of the pains during this stage. You will find, that, however severely they may commence, they last but a short time, and the effect on the os uteri is comparatively slight. If these short, though severe pains, be contrasted with the long-continued and powerful pains which follow them when the liquor amnii is discharged, and the os uteri dilated, the difference in the effect will be sufficiently obvious. As a means, therefore, of conveying the whole muscular power of the uterus upon the os uteri—of moderating and equalizing the force employed—of dilating the mouth of the uterus without exciting irritation—the liquor amnii is of essential importance.

"We shall now consider *the order* observed by the uterus in the contractions which take place. This may be ascertained experimentally. For instance: when the hand is passed into the uterus after delivery, to remove the placenta, we find that it may remain for sometime in the cavity without exciting its contraction, but the moment the hand is being withdrawn, the fundus instantly contracts, and as it passes along the vagina, the contractions are continued from above downwards; so also, in other instances, when the os uteri is only irritated by the fingers of the hand introduced in the vagina, and an attempt is made to dilate it, the fundus immediately contracts, not the os uteri. You have thus a very favorable illustration of the reflex nervous function. Hence we infer that the order of uterine contractions is from the fundus downwards, and that the action is commenced there."

But as this view of the matter has been controverted, Dr. Murphy exhibits at length the reasoning upon which the opposite opinion rests. Wigand is the chief advocate of this doctrine, and his views are copied from the work of Dr. Rigby, as follows:—

"In examining the course of a true pain, we shall find the contractions of the uterus *do not begin in the fundus, but in the os uteri*, and pass from one to the other. *Every pain which commences in the fundus is abnormal;* and either arises from some derangement in the uterine action, or is sympathetic with some irritation not immediately connected with the uterus, as from colic, constipation, etc. We very seldom find that a contraction of the uterus which has commenced in the fundus, passes into the cervix and os uteri, and becomes a genuine effective pain; usually speaking, the contraction is confined to the circumference of the fundus, without detruding the fœtus at all. When a genuine pain comes on, so far from the head being pressed against the os uteri, it at first rises upwards, and sometimes gets even out of reach of the fingers, *whilst the os uteri itself is filled with the bladder of membranes;* if it had commenced in the fundus, instead of the inferior segment of the uterus, so far from the head being drawn up at the first coming on of the pain, it would have been forcibly pushed down against the os uteri. In the course of a few seconds, the contraction gradually spreads over the whole uterus, and is felt especially at the fundus; the head which has been raised somewhat from the os uteri, is now again pushed downwards to it, and seems to act as a wedge for the purpose of dilating it; it is not until the whole uterus is beginning to contract, that the patient has a sensation of pain. We may therefore, consider that a genuine uterine contraction consists of certain phenomena which occur in the following order:—First, the os uteri grows tight, and the presenting part rises somewhat from it, then the rest of the uterus, especially the fundus, becoming hard, the patient has a sensation of pain, and the presenting part of the child advances."

Upon which our author makes the following remarks.

"Now, if we desired an additional evidence to prove that the fundus was the first part of the uterus to contract, and not the os uteri, we could not have a stronger proof than that advanced by Wigand to support a contrary opinion—viz., the head when the contractions commence, getting, "even out of reach of the fingers, whilst the os uteri is filled with the bladder of membranes." In Wigand's explanation, the influence of fluid pressure seems to be altogether forgotten. The immediate effect of contraction commencing at the fundus would be to compress the liquor amnii which of necessity forces its way before the head, on to the mouth of the uterus. The fluid in this position re-acts against the head wit the same power that it is compressed, and therefore pushes it up until the increasing contraction of the fundus forces the head down again, so that you perceive the phenomena quoted are quite consistent with the statement that uterine contraction begins at the fundus; in fact, it could not be otherwise, so long as the waters remain in the uterus. But if the contraction commenced from below, the fluidmust be driven upwards towards the funds, and that portion between the os uteri and head pressed aside, at least in the first instance so that the head might be easily felt when the pain commences, although not so afterwards; but the reverse is the case, and you will find that in those cases where the liquor amnii is in large quantity, that it is difficult to feel the head at all, except in the interval of the pains. "The tightening of the os uteri," alluded to by Wigand, seems to be another source of error on this point, it being generally confounded with muscular contraction of the os uteri. It seems to me to be produced by the pressure of the fluid downwards against the sides of the uterus, combined with the increased determination of the blood towards the os uteri, which arises from the vessels at the fundus expelling a portion of their blood during its contraction. The os uteri is rendered fuller, and the lips are more closed than before; hence the opinion that it is muscular contraction, the evidence for which does not seem to me sufficient to establish so important a fact."

We shall not follow him in his criticism of the various theories advanced, by Dewees and others, to account for this process, but proceed to matters of greater practical moment.

Our next extract is from the sixth lecture, on the second stage of labor, in which are stated, concisely and in a pleasing style, the "obstetric duties of the practitioner." He says:

"The *first object*, then of a vaginal examination in this stage, *is to determine the proportion between the head and the pelvis.* For this purpose the fingers should be passed carefully between both in the interval of the pains, directing them, in the first instance, between the pubis and the head, and moving them round on either side. The ear can be felt if there be sufficient space for the head to pass, but if the head be high up in the pelvis, the finger can only just touch it. If the ear cannot be reached readily, and there seems to be a want of proportion between the head and pelvis, you have still another means of testing its degree, by examining the presenting part of the head. When it is only slightly compressed, the scalp is simply folded or puckered by the closing of the sutures; as the compression increases, these folds merge gradually into one, which ultimately forms a distinct tumor. This continues to enlarge, so that in cases of impaction of the head, it is sometimes of great magnitude. The manner in which this change takes place, and its degree, is generally a sufficient proof of the amount of the disproportion. If the tumor form very slowly, and never increase to any great size, you may infer that the head will pass safely through the pelvis; but if, on the contrary, it increase rapidly, and attain a great size, the indication must be unfavorable.

"The *second object* of a vaginal examination is to ascertain the exact position of the head. We have already pointed out to you the means of distinguishing the different positions from each other. We shall, therefore, at present only bring before your notice those positions which we are directed to by some authors to alter as soon as they are found out, in order to prevent the head from becoming impacted in the pelvis afterwards.

"One of these cases is when the head enters the brim in the left fronto-cotyloid position. It is assumed that this cannot pass safely, but will cause great delay and difficulty in the labor; therefore, it is laid down that the correction must be made the moment the position is ascertained. We have already stated to you the experience of Naegele, confirmed by other observers, that nature, if left to herself, will correct this deviation, by rotating the head into the right occipito-cotyloid position. The probability is, therefore, that by meddling too soon you may prevent this, and prematurely force the head into a more unfavorable position than it had been. The moment this position is detected is not therefore, the time for interference; it is more advisable to wait and observe the course the head will pursue. It may correct itself; it may advance and be delivered in the third position without injury; it may be arrested. The last is the only condition which would justify your aid. The head may then be displaced from its situation, and pressed back in the intervals of the pains, and a *very slight rotation* is generally sufficient to make it glide easily in its proper direction when the pains return. The very same observation applies to those instances where the head and hand, or even arm, descend together. This accident is often the result of the pelvis being too wide, and if so, both will be expelled without difficulty; but sometimes the arm comes down a little too much and prevents the head advancing, or the head may be arrested by the hand descending with it. In either case, the hand or arm can be very easily pushed back when the pain ceases, and so maintained until the next pain advances the head, which generally passes down very rapidly as soon as the correction has been made. When the head is in the cavity of the pelvis, there is not the same danger of displacing it as when it is only entering the brim; and consequently, our previous observations on this accident (pp. 98–99) do not apply to the present case. You should not, therefore, when these deviations occur, too hastily assume that the head cannot be delivered It is more advisable to wait until they become causes of delay.

"The *third object* of a vaginal examination is, to note the progress which the head makes. In natural labor, where no difficulty presents itself, a very few examinations, at proper intervals, will be sufficient for the purpose, because its advance is generally quite obvious; but in difficult labors, where the head makes a very slow progress, and there are other causes of embarrassment present, more care is required; their consideration, however, is beside our immediate subject. Having ascertained the position of the head, and its relation to the pelvis, the next object of attention is its descent upon the perinæum. You must, therefore, *be prepared to give the perinæum support* the moment it suffers any degree of distension. The mode of doing so, which I have been in the habit of adopting, is somewhat different from that directed by the more popular writers on midwifery. Ramsbotham and Rigby both employ the left hand to press against the perinæum, and the right is kept in reserve to make any necessary correction. Churchill and others adopt the same plan. Dr. Rigby admits that 'it is awkward at first, because it requires the hand to be considerably twisted, and makes the wrist ache a good deal.'"

Other directions which will be found by the young practitioner of great service are thus clearly given. The author is still speaking of the mode of supporting the perinæum.

"The plan which I have found the most useful and convenient to adopt at this period of labor, is the following:—To sit behind the patient as she lies upon her left side, the back of the chair being towards the head of the bed, and while the head of the child is passing through the pelvic cavity, to press moderately with the left hand over the hip of the patient. Counter-pressure in this way employed is generally grateful to her, and seems to give her some relief; it assists also in keeping the pelvis fixed when the head is passing the perinæum, the most important part of this process. Having the left hand so employed, the right can be used to support the perinæum, a single fold of a fine napkin should be placed along the edge of the perinæum, and the right hand so applied that the fold of skin between the fore-finger and thumb should correspond to this, the fore-finger and thumb passing on either side of the vulva, and the palm of the hand, resting against a thicker fold of the napkin, applied to the posterior part of the perinæum. By this means you have full power to make any counter-pressure with the palm of the hand which may be necessary, and the fingers being quite close to the edge of the perinæum and vulva, you can easily trace the margin of the perinæum, and feel the head, if necessary. Thus one hand fulfils the office generally assigned to two, and enables you to grasp with the left hand the pelvis, to prevent the patient moving away too suddenly when severer pains come on. If, the head being expelled, this be no longer necessary, you can employ the same hand to support the uterus during its contraction in expelling the body of the child. Besides these advantages, it is certainly less fatiguing. The only inconvenience of this method is, that when the funis is coiled round the neck of the child, so as to make it necessary to remove it, or that the delivery of the shoulders should be assisted, the hands must be changed, that the left may support the perinæum and the right make the required correction. But this is a temporary disadvantage and only arises occasionally. We shall suppose you, therefore, thus prepared to give the perinæum the required support; the only question is, when your assistance is needed. The young practitioner, fully impressed with the importance of preventing laceration, hardly ever commits the mistake of being too late in attending to this point. He very generally errs on the other side: he presses against the perinæum a great deal too soon, and causes unnecessary heat and irritation in consequence, which rather retards its distension. His mistake arises from supposing the perinæum in danger the moment the head touches it. We have explained to you that the head alternately touches and retreats from the perinæum, often for a long time before the perinæum suffers any dangerous distension. You must not, therefore, be too precipitate; it is better to wait until you feel the head protruding, with each pain, through the vulva, because at this time it is getting gradually upon the ischio-pubic ramus, against which it rests, while the anterior part of the head presses, with considerable force, against the perinæum. Caution is also necessary as to the manner in which the perinæum is supported. The object in view is to obviate the effects of too violent distension. The pains at this time are very unequal, sometimes weak and again very strong; you support the perinæum against the latter by moderate counter-pressure, to prevent accidents; but against the former no such precaution is necessary: you must not, therefore, press with every pain indifferently, but only when the uterus is acting with great force. Again: when the head is nearly protruded through the vulva, anxiety to save the perinæum may be the cause of its rupture. For instance, if you attempt to draw the perinæum back over the head, it will be stretched too suddenly over the bi-parietal measurement, the widest part of the head. If, on the other hand you push the head too much forwards, pressing with the pains from the sacrum towards the pubis, the same effect will be produced in a different manner; you force the parietal portion on the head too rapidly through the vulva. At this point it is better to continue the same moderate counter-pressure, to make no attempt to hasten the delivery, and to allow the head to pass along the hollow of the hand, in the same manner as it moved along the curve of the sacrum. When the head is passing out of the vulva, you should then direct it forwards toward the pubis; and when it is delivered, examine carefully lest *the funis may be coiled round the neck.* If such be the case, and that is only a single coil, it will generally be sufficient to draw down a little more of the funis, and loosen it. A single coil seldom retards the delivery of the child, or arrests the fœtal circulation; but two, and even three, coils are sometimes met with, and the child placed in great danger of strangulation. In these cases, as much of the funis as possible should be brought down, and the coils so loosened that one may be drawn over the head. There are cases where this cannot be done, and the only resource left is to tie and divide the funis, and extract the child as soon as possible, in order that respiration may be established. This operation is hazardous to the child's life, and can only be viewed as the lesser of two evils."

From these passages the reader will be able to form his own opinion respecting the style in which the author imparts his precepts. We have no fears that it will differ materially from the one which we have ventured to express.

Having presented these specimens from the body of the work, we shall now copy some of the "*Aphorisms*" with which it is concluded. These aphorisms are a summary of the principles and rules laid down in the lectures. The condensed form given to these important rules in this Summary will be very acceptable to the young practitioner, who may have occasion to refer to a work on obstetrics in the midst of pressing professional duties.

"*Management of Natural Labors.*—The 'show' is a slight sanguineous discharge, arising from the ruptured vessels of the mouth of the uterus, when it first dilates. As soon as this takes place, labor properly begins. The patient has now entered upon the first stage.

"When called upon to attend a patient, *the summons should be instantly responded to.* Your introduction should not be abrupt; and if called to a patient for the first time, still greater caution should be exercised.

"During the early part of the first stage, it is not necessary nor advisable to remain in the room with your patient. It will be sufficient to remain in an adjoining room, until there is occasion to make a vaginal examination.

"When the pains have continued for some time regular and frequent, an examination may then be made. The patient, loosely attired in her night-dress, should lie on the bed on her left side, as near to the edge as possible, having the knees drawn up towards the abdomen. You should then wait until the pain returns, and when it is about to cease pass the fore-finger of the right hand, anointed with cold cream, or any unctuous substance, within the vagina. Examine whether it be rigid or relaxed, dry or moist. Examine the rectum through the posterior wall, and fundus of the bladder through the anterior. Introduce the middle finger, in order to examine the os uteri. Pass both fingers along the sacral side of the vagina, and when you cannot advance them further, direct them forward towards the pubis; you then feel what seems to be the irregular folds of a flaccid bag, projecting into the vagina: examining this with caution, the edge of the os uteri may be traced; if the finger be passed within it, you will sometimes feel the head; you may often fail, and yet the head present. The dilatability of the os uteri, its direction, and the degree to which it is opened, should be ascertained.

"If the membranes are ruptured early, an examination should always be made, lest the funis or the shoulder present. Before the fingers are withdrawn from the neighborhood of the os uteri, examine the distance of the sacrum; and as they are being withdrawn, ascertain if possible the space in the pelvic cavity.

"In order to make a sufficiently careful examination, a little time may be required, during which the pains may return; you should then cease until they subside, noting only those points which have been mentioned; but make it a rule not to withdraw from your examination until you have perfectly satisfied yourself as to the character of the labor. Having accomplished this, a second examination during this stage, unless it be prolonged, would be unnecessary.

"The first stage of labor is not always completed before the second begins. The grinding pains merge into the bearing pains so gradually as to require some attention to observe the change. When the bearing pain comes on, the patient is obliged to grasp firmly whatever is within her reach, she retains her breath mor than before, and sometimes makes an involuntary effort to bear down. Her voice also alters, its tone is more subdued, and she seems more patient of her sufferings than before. Sometimes the complete dilatation of the os uteri is marked by the constitutional symptoms; there may be a slight rigor or vomiting, perhaps a strong inclination to go to stool.

"The patient must now remain in bed, which should be properly prepared for her reception. A skin of morocco leather, or a broad piece of India-rubber cloth, must be placed next the bed, to protect it from being stained, and a blanket folded very wide, and enclosed in a soiled sheet, placed underneath the hip of the patient, as she lies upon her left side. They should be so fastened together, that the whole may be moved at once.

"When the bearing pains become decided, a sheet should be fastened to the bed-post, and so within reach of the patient that she may grasp it firmly. The nurse should support her feet by pressing a pillow against them. Small pillows between the knees are very inconvenient.

"During the second stage, the patient often becomes fatigued and thirsty. You must imperatively forbid heating drinks of all kinds to be given to the patient. A free ventilation of the apartment should be secured, and as few persons as possible permitted to remain in the room.

"In the second stage of labor more than one examination is necessary, but if examinations be repeated too often, the passages become dry and irritated. *The first object* of the examination is to determine the proportion between the head and the pelvis; *the second object* is to ascertain the exact position of the head; *the third object* is to note the progress made by the head.

"In order first *to determine the proportion*, the finger should be passed, in the interval of the pains, between the pubis and the head, and moved round on either side. The ear can be felt if there be sufficient space for the head to pass, but if the head be high up in the pelvis, the finger can only just touch it.

"If the ear cannot be reached, and there seem to be a disproportion, its degree may be judged by feeling the scalp. When the head is only slightly compressed, the scalp is simply folded or puckered by the closing of the sutures, as the compression increases, these folds merge into one, which ultimately forms a distinct tumor. This continues to enlarge, so that in cases of *impaction* of the head it is sometimes of great magnitude.

"*To ascertain the exact position of the head*, pass the finger to the sagittal suture. This traverses the presenting part of the head, and looks towards the sacrum. Direct it along this suture towards the left obturator space and plane of the ischium, a fontanelle will then be felt: if it be the posterior (a small triangular space or point where three sutures meet), it is the first position; if it be the anterior fontanelle (a large membranous space bounded by four sides or undefined), it is the third position When the fontanelle is on the right side of the pelvis, the second position may be distinguished from the fourth in the same manner.

"If there be a doubt of, or difficulty in, feeling the fontanelle, the ear will determine the side of the pelvis to which the occiput is applied, the lobe of the ear being felt on the corresponding side.

"You must be prepared to support the perinæum the moment it suffers any degree of distension. Sit behind the patient as she lies upon her left side, the back of the chair being towards the head of the bed, and while the head of the child is passing through the pelvic cavity press moderately *with the left hand* over the hip of the patient. *With the right hand* support the perinæum. A single fold of a napkin should be placed along the edge of the perinæum, and the right hand so applied that the fold of the skin between the fore-finger and thumb should correspond to it, the fore-finger and thumb passing on either side of the vulva, and the palm of the hand resting against a thicker fold of the napkin, applied to the posterior part of the perinæum.

"The perinæum should not *be pressed against too soon;* rather wait till you feel the head protruding with each pain through the vulva. The pains are very unequal. and the object of being prepared to support the perinæum early is to resist too violent distension by them. Support the perinæum against the powerful pains. It is not necessary to press strongly when the pains are weak.

"When the head is passing the vulva, do not draw the perinæum backwards nor push the head too rapidly forwards In either case the widest part of the head would be forced too soon over the perinæum. When the head is delivered, examine carefully lest the funis be coiled round the neck. If it be a single coil it is sufficient to draw down more of the funis and loosen it. If two or more, one must be brought over the head. When this cannot be done the funis must be divided, which should never be done if it be possible to avoid it.

"When the shoulders are passing the perinæum, caution must be used lest the arms should lacerate it, especially in second positions of the head. Sometimes the shoulders require to be assisted, which may be done by placing the forefinger of the right hand within the axilla of the child's arm on the pubic side, and guarding the perinæum carefully with the left hand.

"As soon as the shoulders and thorax of the child are delivered, it can respire, and is so far beyond danger; no haste should, therefore, be used in extracting the body and lower limbs; it is preferable to allow the uterus gradually to expel them, and while it is doing so, the left hand should be immediately applied over the fundus, in order to maintain a moderate pressure upon the uterus while it is descending towards the pelvis.

"Pressure should then be maintained either by a temporary bandage or by the hand of an assistant, and the funis tied and divided. Apply a strong ligature of housewife thread, bobbin, or narrow tape, about two inches from the umbilicus, and a second about an inch further. You must be careful to see the part of the funis you are dividing, lest the fingers or any part of the child should be in your way, and also to examine the cut surface of the umbilical portion. The blood should be squeezed out of the vessels, and the surface wiped with a napkin, for the purpose of detecting any oozing of hemorrhage that might take place if the funis were not properly tied.

"The hand may be applied to the fundus and a very moderate pressure is often sufficient to expel the placenta completely out of the vagina; but if not, it can be drawn out by the funis quite easily, directing the traction in the axis of the vagina. But if the uterus should not obey the stimulous at first, do not persevere; it is always more advisable to wait for some time than too use too much irritation. Neither should you attempt to remove the placenta by the funis alone.

"After delivery, a bandage is necessary to support the pelvis by compressing it as much as possible, and to support the uterus by moderate and equable pressure over the whole abdomen. The bandage should be rather more than a yard long and half a yard wide. It should first be drawn evenly over the pelvis; its lower edge, when so placed, being about an inch below the trochanter. This margin should be drawn as tightly as the patient will bear and pinned securely below the right trochanter. The bandage should be again drawn and pinned in a similar manner across the crista of the ilium, so that the pelvis may be embraced by this portion of the bandage, about three inches in width, as tightly as possible. The remainder of the bandage should be drawn and pinned with moderate tightness, but equally from the pelvis to the diaphragm, so that the whole abdomen be included in it without projecting over the bandage.

"When the bandage is applied, the folded sheets that had been placed under your patient during labor should be removed and replaced by others dry and warm, in order that she may be induced to sleep. She must not be disturbed afterwards.

"If the placenta be not separated in the first instance, wait for an hour, and again by pressure, excite the action of the fundus. This should be brought as nearly as possible toward the centre of the pelvis, and grasped firmly by both hands. As soon as the fundus becomes hard, strong pressure upon it is generally sufficient to cause the expulsion of the placenta: if it should not, do not use any violence; rather let the nurse, under your direction, maintain the fundus in the same position, while you pass the fingers, and, if necessary, the hand, into the vagina' in order to stimulate the uterus to contraction."

A similar section is devoted to Difficult labors, and another to Obstetric operations.

These Lectures were published before Etherization was introduced into obstetric practice, and of course we have nothing from the judicious author on that subject. A new edition will no doubt be called for in a short time, and in that we may expect an expression of his opionion relative to the value and safety of the new agent.

The mechanical execution of this work is good. The wood cuts are numerous, and will aid the student essentially in mastering the text.

